# Crystal Structure of the Metallo-Endoribonuclease YbeY from *Staphylococcus aureus*

**DOI:** 10.4014/jmb.2209.09019

**Published:** 2022-11-23

**Authors:** Jinwook Lee, Inseong Jo, Ae-Ran Kwon, Nam-Chul Ha

**Affiliations:** 1 Research Institute of Agriculture and Life Sciences, Center for Food and Bioconvergence, Department of Agricultural Biotechnology, CALS, Seoul National University, Seoul 08826, Republic of Korea; 2 Department of Beauty Care, College of Medical Science, Daegu Haany University, Gyeongsan 38610, Republic of Korea; 3 Current address: Infectious Diseases Therapeutic Research Center, Korea Research Institute of Chemical Technology, Daejeon 34114, Republic of Korea

**Keywords:** *Staphylococcus aureus*, YbeY, metalloprotein, endoribonuclease, zinc, crystal structure

## Abstract

Endoribonuclease YbeY is specific to the single-stranded RNA of ribosomal RNAs and small RNAs. This enzyme is essential for the maturation and quality control of ribosomal RNA in a wide range of bacteria and for virulence in some pathogenic bacteria. In this study, we determined the crystal structure of YbeY from *Staphylococcus aureus* at a resolution of 1.9 Å in the presence of zinc chloride. The structure showed a zinc ion at the active site and two molecules of tricarboxylic acid citrate, which were also derived from the crystallization conditions. Our structure showed the zinc ion-bound local environment at the molecular level for the first time. Molecular comparisons were performed between the carboxylic moieties of citrate and the phosphate moiety of the RNA backbone, and a model of YbeY in complex with a single strand of RNA was subsequently constructed. Our findings provide molecular insights into how the YbeY enzyme recognizes single-stranded RNA in bacteria.

## Introduction

RNA molecules are synthesized by RNA polymerases using DNA as a template and processed or degraded by diverse ribonucleases. Regulation of mRNA stability by ribonucleases is essential in adapting bacteria to changing environments [1-3]. YbeY is an endoribonuclease specific to the single-stranded RNA of ribosomal RNAs. Early studies revealed that YbeY was involved in late-stage 70S ribosome quality control and the maturation of the 3' terminus of the 16S rRNA in bacteria [4, 5]. YbeY also plays a key role in the adaptation process of cells by directly modifying mRNAs or processing small RNAs (sRNAs), which regulate mRNA translation and stability [6]. Transcriptomic analyses of the opportunistic gram-negative bacterium *Pseudomonas aeruginosa* revealed that the deletion of YbeY led to the downregulation of catalase genes and increased the susceptibility to oxidative stress. A subsequent study further revealed the pleiotropic roles of YbeY and YbeY-mediated regulation in adapting to environmental stress, which was associated with bacterial virulence [7, 8].

The crystal structure of YbeY from *Escherichia coli* was determined at a resolution of 2.7 Å by a structural genomics consortium group before the function of YbeY was known [9]. The structure showed that YbeY binds Ni^2+^ and coordinates three histidine residues and presumably one water molecule in a tetrahedral geometry. Ni^2+^ was obtained from the purification step using Ni-NTA resin. A later study revealed that Zn^2+^ is required for the nuclease activity of YbeY [4, 5, 10].

*Staphylococcus aureus* is a gram-positive bacterium and has many different genes from gram-negative bacteria. In addition, *S. aureus* contains YbeY, which shows only 29% sequence similarity to *E. coli* YbeY. In this study, we noted the sequence divergence of YbeY from gram-positive bacterial YbeY relative to that from gram-negative bacteria. Furthermore, we determined the crystal structure of YbeY from *S. aureus* (*Sa*YbeY) in complex with a zinc ion at the active site. We then performed biochemical analyses to investigate the molecular mechanisms of YbeY based on the crystal structure of *Sa*YbeY.

## Materials and Methods

### Construction and Protein Expression

The DNA sequence coding the YbeY protein from *S. aureus* (NCBI reference sequence: BAB57732.1) was amplified from the genomic DNA from *S. aureus* Mu50 using PCR. Next, the PCR products and the pET28a vector (Merck) were digested by the restriction enzymes NcoI and XhoI, which were each ligated by T4 ligase, resulting in the pET28a-YbeY plasmid.

### Protein Overexpression and Purification

The synthesized pET28a-YbeY plasmid was transformed into *E. coli* BL21 (DE3), and BL21 cells were cultured at 37°C in 3.0 L of LB medium, to which 100 μg/ml ampicillin was added until the OD_600_ value reached 0.6. For the expression of the YbeY protein, 0.5 mM IPTG was supplied to the LB medium, and after further culturing at 30°C for 6 h, the cells were harvested by centrifugation at 1 400 ×*g* for 7 min at 4°C. Then, the harvested cell pellet was resuspended in 50 ml of cell resuspension buffer containing 20 mM Tris (pH 8.0), 150 mM NaCl and 2 mM β-mercaptoethanol. Next, the resuspended cells were disrupted using a sonicator, and then the cell debris was removed by centrifugation at 20 000 ×*g* for 30 min at 4°C.

The cell lysate was mixed with Ni-NTA agarose resin (1 ml; Qiagen, Germany) in a 2.5 × 10 cm Econo-Column (Bio-Rad) and rolled for 45 min at 4°C. After washing the resin with wash buffer (250 ml) containing 20 mM Tris (pH 8.0), 150 mM NaCl, 20 mM imidazole (pH 8.0), and 2 mM β-mercaptoethanol, the protein was eluted by elution buffer containing 20 mM Tris (pH 8.0), 150 mM NaCl, 250 mM imidazole (pH 8.0), and 2 mM β-mercaptoethanol.

The eluted protein was further purified using anion exchange chromatography (Hitrap Q HP, GE Healthcare, USA). The protein solution was eluted by applying a NaCl concentration gradient from 0 mM to 1 M, and protein was obtained in a range of 400-450 mM NaCl. The eluted fractions were gathered in the appropriate range and purified one step further using a gel filtration chromatographic column (HiLoadTM 26/610 Superdex 200 pg; GE Healthcare) with buffer containing 20 mM Tris (pH 8.0), 150 mM NaCl and 2 mM β-mercaptoethanol. Finally, the collected protein was concentrated with a Vivaspin centrifugal concentrator (10 kDa molecular-weight cutoff; Millipore, USA), resulting in a final protein concentration of 11 mg/ml, and then the solution was stored at -80°C until use.

### Crystallization, Data Collection, and Structural Determination

The initial crystallization screening of *Sa*YbeY was conducted using the automated protein crystal screening equipment MOSQUITO (SPT Labtech) at 14°C with a sitting-drop vapor-diffusion method. Next, the screened conditions were optimized with a reservoir solution containing 0.1 M sodium citrate pH 5.5, 40% (w/v) polyethylene glycol 600, and 1 mM zinc sulfate using the hanging-drop diffusion method at 14°C in a 15-well plate.

To collect diffraction data on the proteins, the *Sa*YbeY crystals were transferred to 2 μl Paratone-N, which is a viscous oil for cryoprotectants, and incubated for a second. Then, the crystals were flash-cooled in liquid nitrogen at -173°C for data collection. The datasets were collected at a wavelength of 1.2818 Å on an Eiger 9 M detector in beamline 5C of the Pohang Accelerator Laboratory, Republic of Korea. The HKL-2000 program was used to process, merge, and scale the diffraction datasets [14]. Table 1 describes the data collection statistics. The structure of *Sa*YbeY was determined using the single-wavelength anomalous diffraction (SAD) method with AutoSol in PHENIX [15]. The structure of YbeY was built and refined using COOT and PHENIX refinement software [16, 17]. Statistical information on data collection and refinement is presented in Table 1.

### SEC-MALS

The SEC-MALS experiment was conducted using a gel filtration column (Superdex-200 10/300 GL, GE Healthcare) at the Korea Basic Science Institute (KBSI). The MALS signals were detected by an 18-angle detector and differential refractometer (Optilab rEX, RI detector, Wyatt Technology, USA) in the Wyatt DAWN HELIOS II MALS system. YbeY (1 mg/ml concentration) was loaded into the gel filtration column in a buffer containing 20 mM Tris-HCl (pH 8.0), 150 mM NaCl, and 2 mM ZnCl_2_. Raw data were analyzed by ASTRA 6 software (Wyatt, Wyatt Technology, USA).

### Isothermal Titration Calorimetry (ITC)

The 50 μM *Sa*YbeY protein samples for the ITC experiment were dissolved in buffer containing 20 mM Tris-HCl (pH 8.0) and 150 mM NaCl. The ITC experiments were performed at 25°C by a MicroCal AutoITC 200 (GE Healthcare) at the Korea Basic Science Institute (KBSI). The ITC results were analyzed using single-site mode, and the MicroCal Origin software package (OriginLab) was used for analysis.

### In Vitro Endoribonuclease Assay

A substrate of single-stranded RNA for the endoribonuclease assay was designed by applying the fluorescein (FAM)-carboxytetramethylrhodamine (TAMRA) quenching system [18]. The RNA sequence for a substrate of *Sa*YbeY (5′-FAM-UCCUUACCUUAA-TAMRA-3′) [4] was synthesized by Bioneer (Republic of Korea). We used 8μM *Sa*YbeY and 2 μM substrate with buffer containing 20 mM Tris-HCl (pH 8.0) and 150 mM NaCl in all endoribonuclease assays. Each metal ion and EDTA were added at 1.5 mM according to each condition. The total reaction volume was 100 μl, and it was incubated at 37°C for 30 min. Subsequently, a microplate reader (Varioskan LUX, Thermo Fisher Scientific, USA) measured increased fluorescence at an emission wavelength of 518 nm.

## Results

### Structural Determination and Overall Structure of YbeY from *S. aureus*

**Fig. 1:**
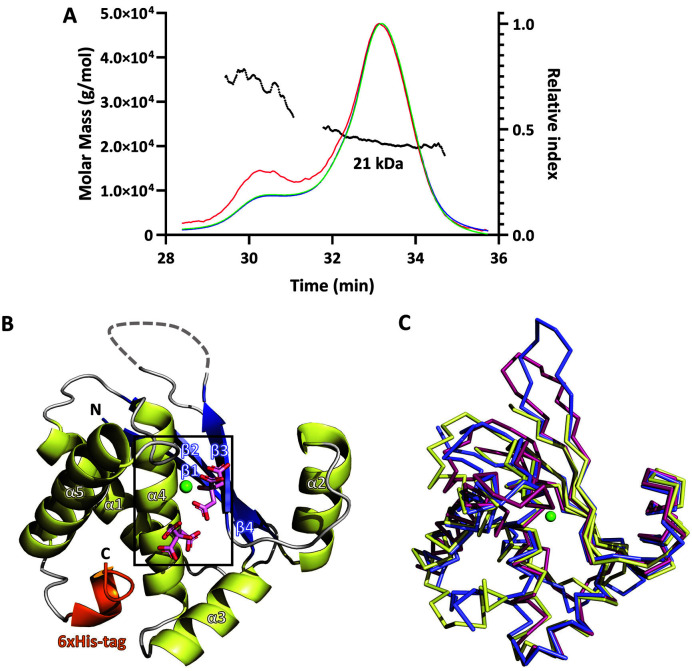
Overall structure of *Sa*YbeY. **A**. SEC-MALS result with the recombinant *Sa*YbeY protein. Superdex 200 Increase 10/300 GL (GE Healthcare) and 18-angle MALS equipment were used. The protein was monitored by the refractive index (RI, green line), ultraviolet light (UV, blue line), and light scattering (LS, red line), as shown on the right Y-axis. The MALS-based molar masses are shown as black dots (left y-axis). **B**. Asymmetric unit structure of *Sa*YbeY shown in cartoon representations. The α-helices are displayed in yellow, the β-strands are shown in blue, and the loop is shown in gray. The N- and C-terminal regions are indicated. The hexahistidine tag is appended to the C-terminal end of *Sa*YbeY (orange). The disordered region is along the dotted line. The Zn^2+^ (green) and citrate ions (pink) are shown in ball-and-stick representations. The black box indicates the active site, which is enlarged in Fig. 2B. Structural superposition of *Sa*YbeY (yellow) on *E. coli* YbeY (blue; PDB code 1XM5) and *A. aeolicus* YbeY (magenta; PDB code 1OZ9) in the Cα representation. The green ball represents the bound Zn^2+^ in *Sa*YbeY.

The *E. coli* YbeY structure was determined before the biological function of YbeY was revealed [9]. The *E. coli* YbeY contained Ni^2+^ in place of the Zn^2+^ binding site since Ni^2+^ was incorporated during purification with Ni-NTA resins. Although the Ni^2+^ coordination environment in the crystal structure seemed to occur in the octahedral geometry, it was not fully determined. In this study, the full-length *Sa*YbeY protein with C-terminal hexahistidine tags was overexpressed in *E. coli*. The SEC-MALS results for the purified *Sa*YbeY protein showed that YbeY, including the C-terminal hexahistidine-tag, has a molecular weight of 21 kDa. This result indicated that *Sa*YbeY behaves as a monomer in the presence of Zn^2+^ in solution (Fig. 1A). To gain structural information on YbeY from *S. aureus* (*Sa*YbeY), we determined the crystal structure of full-length *Sa*YbeY in complex with Zn^2+^ at a resolution of 1.9 Å. The *Sa*YbeY crystal contained one protomer in the asymmetric unit, which did not extensively interact with the neighboring molecules in the crystal. This finding is consistent with the SEC-MALS results.

The *Sa*YbeY structure consists of a four-stranded β-sheet and five α-helices. The fold matching program FoldSeek revealed that the structural homologues to *Sa*YbeY structure in the protein databank include the hypothetical protein AQ_1354 from *Aquifex aeolicus* (PDB code: 1OZ9), which presents a similar protein fold; YbeY from *E. coli* (PDB code: 1XM5); TM1509 from *Thermotoga maritima* (PDB code: 1TVI); and HI0004 from *Haemophilus influenzae* (PDB code: 1XAX). Furthermore, a comparison of *Sa*YbeY with *E. coli* YbeY and *A. aeolicus* YbeY, whose crystal structures were identified, showed that the three proteins exhibited nearly similar structures (RMSD ~0.929 Å between the matched 92 and 92 atoms). However, the long loop connecting the β-strands β3 to β4 was disordered in the *Sa*YbeY structure, which was well ordered in the other protein structures (Fig. 1C).

### Active Site

**Fig. 2:**
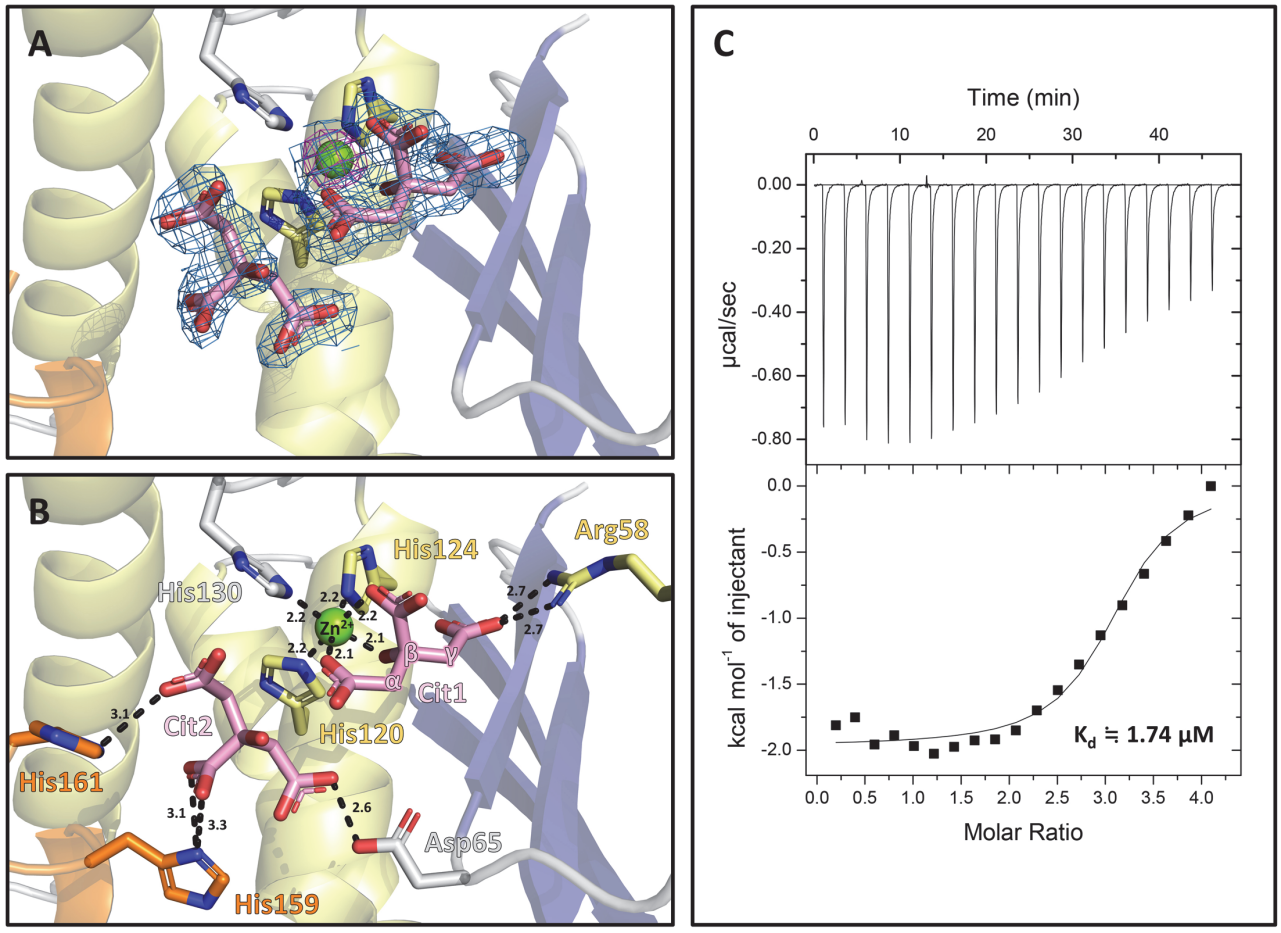
Binding of Zn^2+^ and citrate ions at the active site of *Sa*YbeY. **A**. 2FoFc map of the bound zinc and citrate ions at the active site contoured at the 1.0 σ level (blue line). The bound zinc ion is depicted as a green sphere, around which an anomalous difference Fourier map contoured at 5.0 σ is shown (magenta line). **B**. Microenvironment of the zinc (green) and citrate ions (pink). The residues involved in the zinc and citrate ions are shown in stick representations, where the color code of the residues follows Fig. 1B. The black dotted lines indicate the Zn^2+^-coordination bonds. **C**. Isothermal titration calorimetry (ITC) result of *Sa*YbeY against Zn^2+^. The raw data from ITC are shown in the upper panel, and measured heat is shown in the bottom panel. The best fitting line and displayed dissociation constant (K_d_) were calculated by MicroCal Origin software.

The active site cleft was identified between the protruding α5 and α2 from the flat surface of α3 (Fig. 1B). The bound Zn^2+^ was found at the active site near α4, which was confirmed by the anomalous signal from the diffraction dataset collected at the Zn absorption edge wavelength (Fig. 2A). The Zn^2+^-binding site was matched to that of Ni^2+^ in the octahedral geometry of *E. coli* YbeY.

Strong extra electron density maps were found near the bound Zn^2+^ in both the Fo-Fc and 2Fo-Fc maps (Fig. 2A). Two citrate molecules (Cit1 and Cit2) were assigned to the electron density maps based on the matched molecular shapes and crystallization solution containing 0.1 M sodium citrate (pH 5.5). The first citrate ion (Cit1) participates in Zn^2+^ coordination. Zn^2+^ is hexacoordinated by the three conserved histidine residues (His120, His124, and His130) of YbeY, α- and β-carboxylic groups and β-hydroxy group of the citrate molecule (Cit1). The binding of the Cit1 molecule is further stabilized by the ionic interaction with Arg58 from α-helix 2 (Fig. 2B).

The second citrate ion (Cit2) appeared to be bound more loosely than the first citrate ion. The second citrate ion is bound by the carboxylic groups of the Asp65 side chain and the three histidine residues: His130 in the loop (α4-α5) and the two histidines in the hexahistidine tag (Fig. 2B). Since two histidine residues in the hexahistidine tag were used for purification, the second citrate ion is likely not bound to native YbeY. However, the hexahistidine tag was ordered at the bottom region of the putative substrate-binding region near the bound Zn^2+^. The hexahistidine tag was in the α-helical conformation and the loop conformation in the C-terminal end of *Sa*YbeY, thus occupying the putative substrate-binding space between α3 and α5 (Fig. 1B).

To estimate the binding affinity of zinc and citrate ions to *Sa*YbeY, we performed isothermal titration calorimetry (ITC) experiments. The results indicated that Zn^2+^ is bound strongly and presents a dissociation constant (K_d_) of 1.74 μM (Fig. 2C). However, the citrate ion did not bind tightly enough to measure the binding affinity to *Sa*YbeY in the ITC experiment (Fig. S1). The high concentration of citrate ions during the crystallization process likely promoted the binding of citrate, which provided structural insights into how the negatively charged molecules are bound and coordinate the metal ions at the active site of YbeY.

### Zinc-Dependent Activity of YbeY

**Fig. 3:**
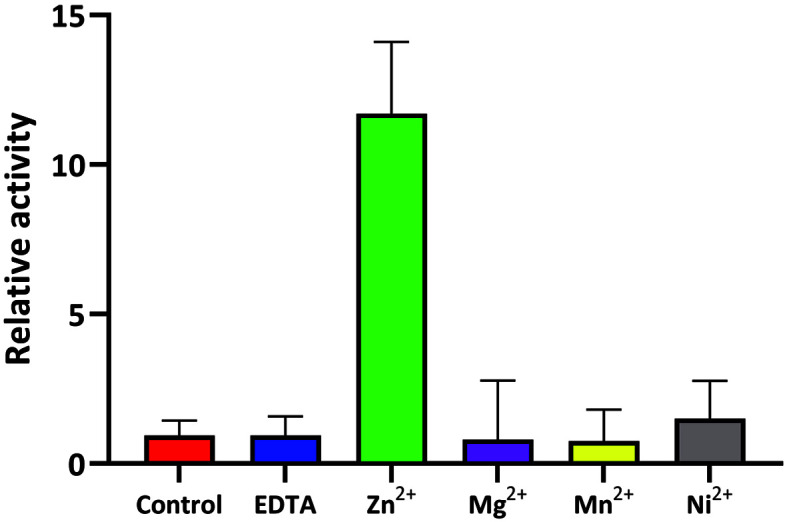
Endoribonuclease activities of *Sa*YbeY in the presence of diverse metal ions. FAM-TAMAR-tagged RNA substrates were used in this study. The endoribonuclease activity of the *Sa*YbeY protein degrades the RNA substrate, resulting in fluorescence from FAM by cleaving off the TAMRA from the substrate. In the x-axis, control represents the basal level of the *Sa*YbeY activity in the buffer without metal ions, and others represent activity with each additive. The relative activity of *Sa*YbeY (y-axis) was measured based on the increased amount of fluorescence. The assay was conducted two times to plot the mean with a column graph, and the error bar is represented at the top of each column.

As YbeY shows metal-dependent endoribonuclease activity [11], we tried to determine the metal ion that is required for the enzymatic activity of *Sa*YbeY. A single-stranded RNA with twelve ribonucleotides was used as the substrate of YbeY, and the endoribonuclease activity of *Sa*YbeY was measured using the FAM-TAMRA fluorescence quenching system using the two-chromophore-containing substrate. The basal activity of *Sa*YbeY in the buffer without zinc ion was nearly undetectable like in the buffer containing the chelating agent EDTA. However, when Zn^2+^ was added to *Sa*YbeY, the activity increased dramatically (Fig. 3).

To examine the Zn^2+^-specific endoribonuclease activity of *Sa*YbeY, we conducted the same assay by adding different metal ions to the *Sa*YbeY protein: Mg^2+^, Mn^2+^, and Ni^2+^. The results indicated that *Sa*YbeY activity was not detected with the addition of these ions, which is consistent with that observed for the *Sa*YbeY protein without additional metal ions in the reaction buffer. These results indicated that *Sa*YbeY has Zn^2+^-dependent activity, as observed in *E. coli* YbeY.

### Model of Substrate Binding

We noted the structural and charge similarities between the carboxyl group of citrate ions and the substrate single-stranded RNA phosphate group. We excluded the second citrate ion in the modeling since it is unlikely to represent a physiological state of *Sa*YbeY. Instead, we noted the basic residues Arg58 and Lys60 on the surface of the substrate binding region. We modeled the substrate single-stranded RNA on the substrate binding site by superposing the carboxylic groups of the bound citrate molecules and the RNA backbones near the basic residues on the YbeY surface (Fig. S2).

**Fig. 4:**
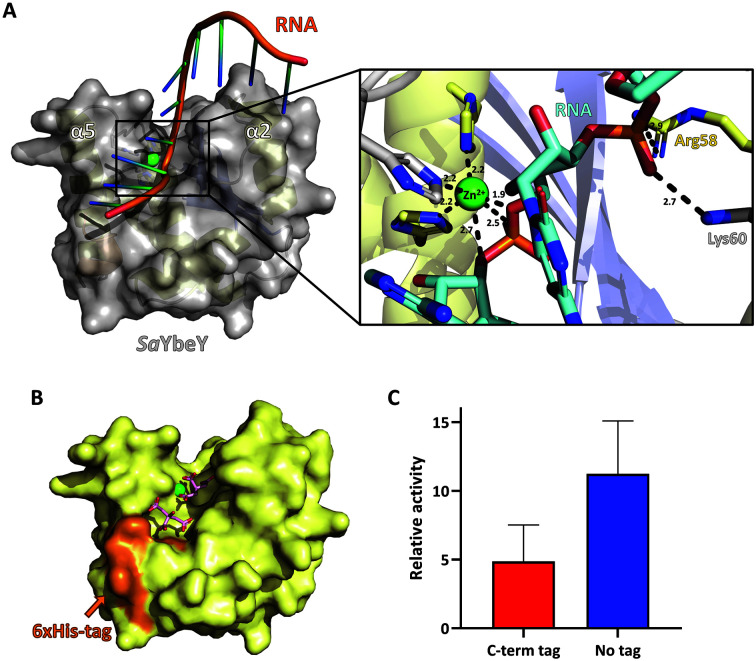
Predicted model of *Sa*YbeY in complex with the substrate single-stranded RNA. The 10-nt RNA used in the crystal structure of GTPase with 16S rRNA [19] (PDB code: 3R9X) was docked on the active site surface of *Sa*YbeY. The substrate binding prediction was conducted by the HADDOCK 2.4 server (Bonvinlab), and the black square represents the active site of *Sa*YbeY, which is enlarged in the right panel. **A**. In the left panel, *Sa*YbeY is displayed in the surface representation (gray), and the docked RNA molecule is in the cartoon representation (orange for the backbone and cyan for the bases). Zn^2+^ is in the green ball, and the active site region is boxed and enlarged in the right panel. At the active site, Zn^2+^ is hexacoordinated by three histidine residues of *Sa*YbeY and three oxygen atoms from the phosphate backbone of RNA. Arg58 and Lys60 make ionic interactions with the phosphate backbone. **B**. Possible role of the C-terminal hexahistidine tag in the *Sa*YbeY structure. A surface representation of *Sa*YbeY is shown (yellow), and the C-terminal hexahistidine tag indicated by the arrow is highlighted orange. **C**. Activity of *Sa*YbeY depends on the presence or absence of a C-terminal hexahistidine tag. The relative endoribonuclease activities (y-axis) of the C-terminal hexahistidine-tagged *Sa*YbeY (C-term tag) and nontagged *Sa*YbeY at the C-terminal end (No-tag).

In the modeled structure, the single-stranded RNA was located in the active site cleft between α2 and α5, and a phosphate backbone connecting nucleotides 3 and 4 was located near Zn^2+^ at the active site of *Sa*YbeY. Zn^2+^ was hexacoordinated by the protein and the phosphate group of the modeled RNA molecule. In addition, the basic residues Arg58 and Lys60 were also located near the RNA backbone, thus showing the possible interactions of *Sa*YbeY with RNA (Fig. 4).

Since the binding of the RNA molecule is slightly interfered with by the C-terminal hexahistidine tag, the hexahistidine tag likely inhibited the activity of *Sa*YbeY (Fig. 4B). Thus, we created the *Sa*YbeY protein without the C-terminal hexahistidine tag and compared the ribonuclease activity to that of *Sa*YbeY with the C-terminal hexahistidine tag, which was used for structural determination. As shown in the results, the C-terminal tag partly inhibited the activity of *Sa*YbeY, thus supporting our substrate-docking model (Fig. 4C).

## Discussion

In this study, we determined the crystal structure of YbeY from *S. aureus* in complex with a zinc ion and two citrate ions. Our structure showed that the zinc ion is hexacoordinated in the octahedral geometry by the three conserved histidine residues and the bound citrate molecule at the active site. Notably, the bound citrate ion acted as the fourth to sixth ligands of the zinc ion in the coordination geometry. We subsequently tested whether only zinc ions activate the ribonuclease activity of *Sa*YbeY. Thus, our Zn^2+^-bound structure showed the active catalytic state of the ribonuclease *Sa*YbeY. Furthermore, the modeled structure with the substrate single-stranded RNA showed that the phosphate backbone coordinates the ligands of the citrate ion with the fourth to sixth ligands of the zinc ion.

Zinc ions are required for the structural integrity and/or catalysis of many proteins [12]. Although zinc ions are found in the octahedral geometry with six ligands (hexacoordination), zinc ions are more frequently tetra-coordinated in the tetrahedral geometry in protein environments. Thus, it is noteworthy that the zinc ion at the active site of *Sa*YbeY is hexacoordinated with the citrate ion and the modeled RNA molecule. Interestingly, Ni^2+^, Mg^2+^, and Mn^2+^, which usually undergo hexacoordination with ligands, interfered with the *Sa*YbeY protein, which is inconsistent with that observed for Zn^2+^. YbeY and its homologues remains elucidated on how zinc is involved during the catalysis. We speculate that the geometry and electrostatic property of the hexacoordinated zinc ion are the most suitable for the catalysis of the ribonuclease activity of *Sa*YbeY. We believe that the interconversion between the hexa- and tetra- coordination of the bound zinc are important in the substrate recognition and the catalysis steps. The unique coordination property of zinc might contribute to the zinc-dependent ribonuclease activity of YbeY although further study is required.

YbeY is the essential gene for the survival of all bacteria, including gram-negative and gram-positive bacteria. However, the functions of YbeY remain ambiguous and need to be studied. YbeY sequences are evolutionarily conserved within both gram-negative bacteria and gram-positive bacteria: 45–70% in gram-negative bacteria and 60–80% in gram-positive bacteria. However, we found a substantial sequence difference between gram-negative and gram-positive bacteria. The YbeY from the gram-positive bacterium *S. aureus* showed only 29% sequence similarity to the YbeY from the gram-negative bacterium *E. coli*. This study revealed that YbeYs showed high structural similarities between gram-negative and gram-positive bacteria, despite the relatively low sequence similarity. In particular, the Zn^2+^-binding environment seems important for understanding the biochemical properties of YbeY in all bacteria. Moreover, since YbeY is also essential in the survival and virulence of the representative pathogenic bacteria *Pseudomonas aeruginosa* and *Vibrio cholerae* [7, 13], this study will also provide structural and functional insights into the development of inhibitors of the pathogenesis of *S. aureus*.

## Supplemental Materials

Supplementary data for this paper are available on-line only at http://jmb.or.kr.

## Figures and Tables

**Table 1:** X-ray diffraction and refinement data.

Data collection	
Beamline PAL	5C
Wavelength (Å)	1.28176
Space group	*P*6_3_
Cell dimensions	
*a, b, c* (Å)	100.7, 100.7, 50.2
Resolution (Å)	50.0-1.90 (1.93-1.90)
*R*_pim_	0.012 (0.044)
*R*merge	0.056 (0.184)
*I/σI*	56 (14.6)
Completeness (%)	96.6 (88.7)
Redundancy	21.1 (18.8)
Refinement	
Resolution (Å)	43.59-1.90
No. of reflections	22298
R_work_/R_free_	0.1967/0.2256
No. of atoms	
Total	1291
Protein	1210
Water	54
Citrate	26
Average B-factor (Å^2^)	
Wilson	17.44
Protein	20.60
Water	23.72
Citrate	25.73
RMSD.	
Bond lengths (Å)	0.007
Bond angles (°)	0.890
Ramachandran plot	
Favored (%)	99.30
Outliers (%)	0.00
PDB code	7Y7O

*The values in parentheses are for the highest resolution shell.
