# Supplementary material for: Crystal Structure of the Metallo-Endoribonuclease YbeY from Staphylococcus aureus
Source: J Microbiol Biotechnol. 2022 Nov 23;33(1):28–34. doi: 10.4014/jmb.2209.09019 (PMC9895993; doi:10.4014/jmb.2209.09019)
Supplement: Supplementary file 1 [file jmb-33-1-28-supple.pdf]

J. Microbiol. Biotechnol.
https://doi.org/10.4014/jmb.2209.09019

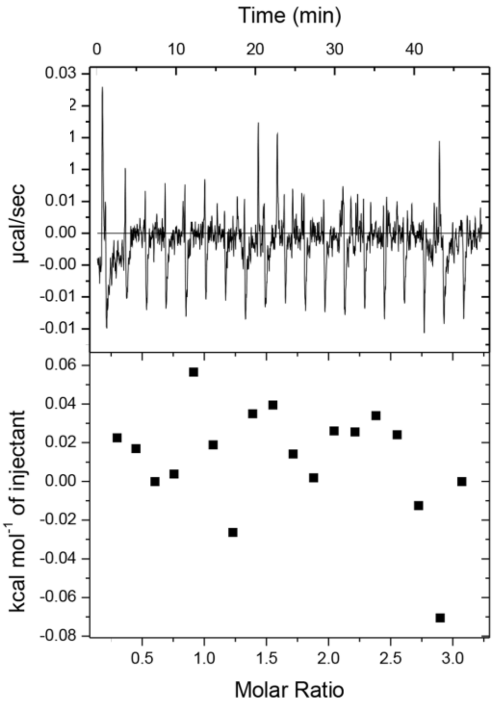

Supplementary Figure 1. ITC results used to analyze the binding affinity between citrate ions and *Sa*YbeY. ITC raw data and generated heat are shown in the upper and bottom panels, respectively.

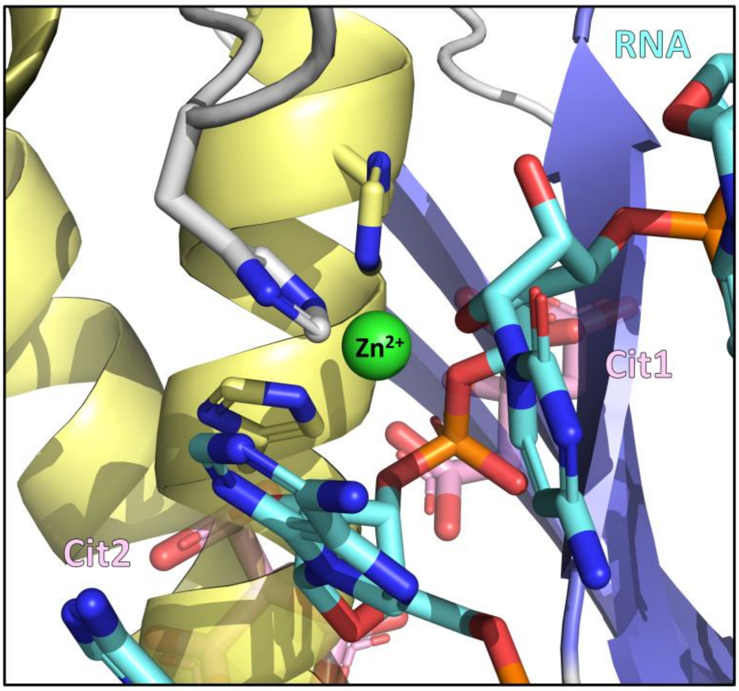

Supplementary Figure 2. Superposition of citrate ions and predicted RNA substrate in the active site of *Sa*YbeY. The secondary structure and residues follow Fig. 2B. The predicted RNA molecule is displayed in a stick representation (orange: phosphate backbone, cyan: nucleobase).

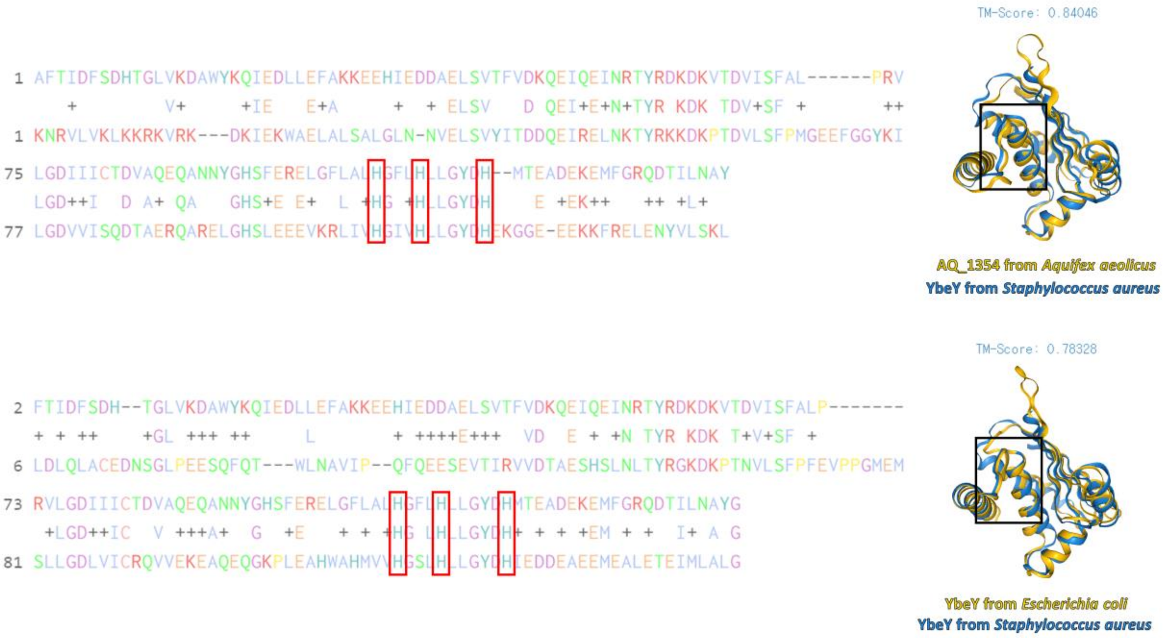

Supplementary Figure 3. Structural-based sequence alignment by FoldSeek. *Sa*YbeY is aligned with AQ_1354 from *Aquifex aeolicus* and YbeY from *Escherichia coli*, respectively. The three zinc-binding histidines are marked with red squares in the left protein sequence panel. The black square in the protein structure alignment represents a zinc-binding site of YbeY.
